# Xuebijing injection inhibited neutrophil extracellular traps to reverse lung injury in sepsis mice *via* reducing Gasdermin D

**DOI:** 10.3389/fphar.2022.1054176

**Published:** 2022-11-16

**Authors:** Ting Shang, Zhi-Sen Zhang, Xin-Tong Wang, Jing Chang, Meng-En Zhou, Ming Lyu, Shuang He, Jian Yang, Yan-Xu Chang, Yuefei Wang, Ming-Chun Li, Xiumei Gao, Yan Zhu, Yuxin Feng

**Affiliations:** ^1^ State Key Laboratory of Component-based Chinese Medicine, Tianjin University of Traditional Chinese Medicine, Tianjin, China; ^2^ Research and Development Center of TCM, Tianjin International Joint Academy of Biotechnology and Medicine, Tianjin, China; ^3^ Key Laboratory of Molecular Microbiology and Technology for Ministry of Education, College of Life Sciences, Nankai University, Tianjin, China

**Keywords:** sepsis-induced lung injury, Xuebijing injection, neutrophil recruitment, neutrophil extracellular traps, Gasdermin D

## Abstract

The mortality of sepsis and septic shock remains high worldwide. Neutrophil extracellular traps (NETs) release is a major cause of organ failure and mortality in sepsis. Targeting Gasdermin D (GSDMD) can restrain NETs formation, which is promising for sepsis management. However, no medicine is identified without severe safety concerns for this purpose. Xuebijing injection (XBJ) has been demonstrated to alleviate the clinical symptoms of COVID-19 and sepsis patients, but there are not enough animal studies to reveal its mechanisms in depth. Therefore, we wondered whether XBJ relieved pulmonary damage in sepsis by suppressing NETs formation and adopted a clinically relevant polymicrobial infection model to test this hypothesis. Firstly, XBJ effectively reversed lung injury caused by sepsis and restrained neutrophils recruitment to lung by down-regulating proinflammatory chemokines, such as CSF-3, CXCL-2, and CXCR-2. Strikingly, we found that XBJ significantly reduced the expressions of NETs component proteins, including citrullinated histone H3 (CitH3), myeloperoxidase (MPO), and neutrophil elastase (NE). GSDMD contributes to the production of NETs in sepsis. Notably, XBJ exhibited a reduced effect on the expressions of GSDMD and its upstream regulators. Besides, we also revealed that XBJ reversed NETs formation by inhibiting the expressions of GSDMD-related genes. Collectively, we demonstrated XBJ protected against sepsis-induced lung injury by reversing GSDMD-related pathway to inhibit NETs formation.

## Graphical Abstract

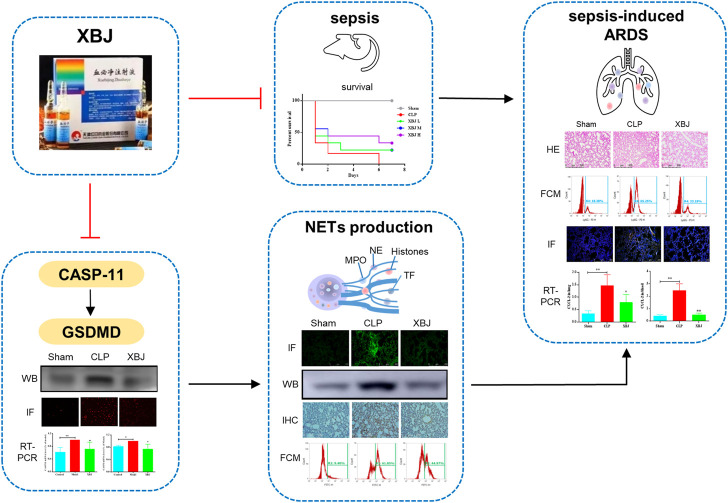

## Highlights

1) XBJ reversed the NETs formation in sepsis-induced lung injury.
2) XBJ decreased the GSDMD expression in lung tissues and neutrophils.
3) XBJ alleviated the NETs formation by inhibiting GSDMD excessive expression.

## Introduction

Sepsis is a life-threatening multiple organ dysfunction syndrome (MODS) caused by the host’s malfunctioning immune response to microbial invasion with unacceptably high mortality, posing a serious threat to human health (Prescott and Angus, 2018). Among multiple organ injuries during sepsis, the lung is the first and most frequent organ to fail, which is also called acute respiratory distress syndrome (ARDS) or acute lung injury (ALI) (Ling et al., 2018). Sepsis is the leading indirect cause of ALI/ARDS in the ICU (Intensive Care Unit). The mortality of sepsis-associated ARDS is higher than that of non-sepsis ARDS (Dickson et al., 2016). Despite numerous technological advances in ICU, mortality from sepsis and systemic inflammatory response syndrome (SIRS) remains high, and no effective therapies are aimed at the syndrome (Silversides et al., 2017). Thus, developing new treatment strategies for sepsis-associated ARDS is of great importance.

Neutrophils is a type of polymorphonuclear leukocyte, that serve as the major initiator of acute inflammation. Under pathological conditions, activated neutrophils release their nuclear contents including histones, myeloperoxidase (MPO), neutrophil elastase (NE), and DNA fragments, which form neutrophil extracellular traps (NETs) (Burgener and Schroder, 2020). Increasing recent evidences reveal that NETs and NE are linked to the activation of coagulation, which contribute to lung injury, causing microvascular circulation disorders and pulmonary edema (Abrams et al., 2019). Neutrophil recruitment within the lung frequently occurs in small capillaries, which is largely dependent on chemokines (Margraf et al., 2019). C-X-C motif ligand type 2 (CXCL-2) has been previously suggested to recruit neutrophils and almost is derived from neutrophils (Girbl et al., 2018). And blocking CXCL-2 and its receptor CXCR-2 prevents mice from lung injury (Margraf et al., 2019).

When sepsis does occur, Gasdermin D (GSDMD) is cleaved following caspase-1/caspase-11 activation, resulting in pores formation, water influx and cell swelling (Kovacs and Miao, 2017). Some researches reveal GSDMD over-expression leads to the formation of pore-induced intracellular traps (Jorgensen et al., 2016). And GSDMD plays a crucial role in the formation of NETs (Chen et al., 2021). In neutrophils, GSDMD is cleaved by serine proteases, like NE, causing the release of granular proteins that are required for NETs production (Sollberger et al., 2018). Overall, GSDMD plays a unique role in promoting NETs formation. Knocking out caspase-11 or GSDMD genes (caspase-11^−/−^ or GSDMD^−/−^) has been shown to protect the animals from septic shock (Hu et al., 2020).

Xuebijing (XBJ) injection is composed of extracts from Radix Paeoniae Rubra (*Paeonia lactiflora* *Pall.*), Szechuan Lovage Rhizome (*Ligusticum chuanxiong Hort.*), Flos Carthami (*Carthamus tinctorius L.*), Angelica sinensis [*Angelica sinensis (Oliv.) Diels*] and Radix Salviae Miltiorrhizae (*Salvia miltiorrhiza Bunge*). It is a compound intravenous injection developed by Professor Jinda Wang of Tianjin First Central Hospital on the basis of Xuefuzhuyu decoction, which is based on the theory of combined treatment of bacteria, poison and inflammation, mainly used to prevent sepsis (Li et al., 2021). In 2004, it was approved by China Food and Drug Administration (CFDA) to introduce into the clinical guideline of SIRS, MODS and sepsis (Chen et al., 2017). As one of the three formulated Chinese medicines for COVID‐19 treatment in China, XBJ has been shown to be remarkably effective in improving patient symptoms with pneumonia (Huang et al., 2021). A number of studies have shown that XBJ can reduce inflammatory cytokines, alleviate coagulation and microcirculation (Shi et al., 2017). The major components of XBJ, paeoniflorin and hydroxysafflor yellow A are identified can down-regulate GSDMD expression in depression and cerebral ischemia reperfusion models, respectively (Tan et al., 2020; Tian et al., 2021). Besides, hydroxysafflor yellow A also can reduce NETs production (Wang et al., 2020). In our previous study, we found XBJ eased a series of pro-inflammatory cytokines production related to neutrophils recruitment in septic mice, such as CXCL-1, CXCL-2, and IL-1β (Wang et al., 2021). Thus, we asked whether XBJ can regulate NETs formation and its related signaling pathway to prevent lung injury in septic shock.

In this work, we found XBJ could protect against lung injury in sepsis, which was achieved by blocking NETs formation. And we further verified XBJ regulated NETs formation by restraining the GSDMD-mediated inflammatory death in neutrophils.

## Methods

### Experimental animals and ethical statement

Research on laboratory animals was conducted according to the internationally accepted principles for laboratory animal use and care in the US guidelines and the guidelines of Tianjin University of Traditional Chinese Medicine Animal Research Center. The protocol was approved by the Tianjin University of Traditional Chinese Medicine Animal Research Committee (TCM-LAEC2022097). All animal experiments were performed with weight-(22–25 g) and sex-(male) matched 8-week ICR mice that were purchased from Vital River Company (Beijing, China). The mice were housed in animal facility free of pathogens, and their overall health status was checked out by trained technicians.

### Cecal ligation puncture model

A murine CLP model was performed following the established protocol (Wang et al., 2021). Briefly, after the mice were anesthetized with tribromoethanol (300 mg/kg), the abdomen was depilated, and the cecum was ligated below the ileocecal valve. The cecum was then punctured once with an 18-gauge needle, and a small amount of feces was squeezed out. Sham mice were subjected to the same procedure, including opening the peritoneum and exposing the bowel. XBJ (batch number: Z20040033) was injected by tail vein with 3 ml/kg (low dose), 9 ml/kg (medium dose) and 18 ml/kg (high dose) for 1 day.

### Hematoxylin and eosin staining

H&E staining was conducted as described (Zhou et al., 2021). Briefly, 24 h after CLP, the mice were euthanized by cervical dislocation, lung tissues were collected and fixed with 4% formalin, then dehydrated and paraffin embedded. After cutting the tissues into 4 μm-thick sections, the sections were stained with H&E and visualized under a microscope.

### Immunofluorescence assay of lung tissues

The paraffin sections of right lung lobes were deparaffinized. After inhibiting endogenous peroxidase activity and repairing antigen, the non-specific binding sites were blocked with 10% bovine serum albumin (BSA) and incubated with primary antibodies including rabbit anti-NE (bs-23549R, Bioss, Beijing, China) and rabbit anti-CitH3 (AF0863, Affinity, Colorado, United States) for 12 h at 4°C and washed with PBST, then staining tissues with Goat Anti-Rabbit IgG H&L (Alexa Fluor®488, ab150077, Abcam, London, UK) and Goat Anti-Rabbit IgG H&L (Alexa Fluor®647, ab150115, Abcam) secondary antibodies, respectively. The expression levels of NE and CitH3 were analyzed with ImageJ software (National Institutes of Health, Bethesda, MD, United States) after being detected by an optical microscope (Vectra 3, PerkinElmer, Waltham, United States) (Liu et al., 2022).

In addition, dewaxed and blocked tissues were incubated with FITC anti-mouse F4/80 antibody (1:50, 123107, Biolegend, California, United States), PE anti-mouse Ly-6G antibody (1:50, 127607, Biolegend) and FITC anti-mouse MPO antibody (1:50, ab90812, Abcam) for 1 h at 37°C, respectively, followed by staining with DAPI (1:200) to label cell nuclei for 10 min at 37°C. The expressions of these genes were detected by optical microscope and quantified using ImageJ software (National Institutes of Health).

### Wet-to-dry lung weight ratio

The left lobe tissues were used to assess the wet-to-dry weight ratio by the gravimetric method at 24 h after CLP. After the wet lungs were weighed, the lung tissues were dried at 80°C for 48 h. Then, the dry lungs were measured and the ratio between wet and dry lung weights was calculated (Wang Y. et al., 2022).

### Evans blue albumin permeability measurement

Evans blue (Sigma-Aldrich, Saint Louis, United States) was dissolved in PBS in a concentration of 0.5%. EBA solution was prepared by adding BSA to 0.5% Evans blue to a final concentration of 4% and then filtered through a 0.22 μm syringe filter. To evaluate the alveolar capillary barrier function, EBA (20 mg/kg) was administered *via* the tail vein injection 1 h before euthanasia. And the whole blood was obtained *via* the eyeball for plasma EBA measurement. The right lung was weighed and stored for subsequent measurement. The lung tissue was homogenized in 2 ml PBS and incubated with additional 2 ml formamide at 60°C for 18 h. Formamide extracts were centrifuged at 15,000 g for 30 min at 4°C and the supernatants were collected to quantify EBA absorbance at 620 nm. EBA permeability index was calculated by dividing the pulmonary tissue EBA absorbance at 620 nm per Gram by the corrected plasma EBA absorbance (Li et al., 2010).

### Real-time PCR

In brief, total RNA was extracted using Trizol assay, which included lysing cells, isolating/precipitating/washing RNA, dissolved with DEPC H_2_O, and reverse transcribed according to the protocol of kit (Transcriptor First Strand cDNA Synthesis Kit, Roche, Basel, Switzerland). The qPCR Core Kit for SYBR Green and the LightCycler 480 II (Roche) were used as the instruction by the manufacturer. A housekeeping gene, glyceraldehyde-3-phosphate dehydrogenase (GAPDH), was used as a measure of relative mRNA levels (Liu et al., 2022). Table 1 presented the primers sequences for real-time PCR, which were synthesized by Sangon Company (Shanghai, China).

**TABLE 1:** The primer sequences for real-time PCR.

Gene	Forward Primer (5′-3′)	Reverse Primer (5′-3′)
GAPDH	TGG​TGA​AGC​AGG​CAT​CTG​AG	TGC​TGT​TGA​AGT​CGC​AGG​AG
CSF-2	GGC​CTT​GGA​AGC​ATG​TAG​AGG	GGA​GAA​CTC​GTT​AGA​GAC​GAC​TT
CSF-3	ATG​GCT​CAA​CTT​TCT​GCC​CAG	CTG​ACA​GTG​ACC​AGG​GGA​AC
CXCL-2	CCA​ACC​ACC​AGG​CTA​CAG​G	GCG​TCA​CAC​TCA​AGC​TCT​G
CXCL-3	AGG​CCC​CAG​GCT​TCA​GAT​AAT	AAT​GCA​GGT​CCT​TCA​TCA​TGG​T
Caspase-11	ACA​AAC​ACC​CTG​ACA​AAC​CAC	CAC​TGC​GTT​CAG​CAT​TGT​TAA​A
HMGB1	GGC​GAG​CAT​CCT​GGC​TTA​TC	GGC​TGC​TTG​TCA​TCT​GCT​G
IL-18	GAC​TCT​TGC​GTC​AAC​TTC​AAG​G	CAG​GCT​GTC​TTT​TGT​CAA​CGA
Caspase-1	ACA​AGG​CAC​GGG​ACC​TAT​G	TCC​CAG​TCA​GTC​CTG​GAA​ATG

### Western blotting

RIPA buffer (Solarbio, Beijing, China) was used to lyse lung tissues, and the supernatant was centrifuged at 13,000 rpm at 4°C for 10 min. BCA Protein Assay Kit (PC0020, TransGen Biotech, Beijing, China) was then used to determine the total protein concentration. After adding the SDS-PAGE loading buffer (P0015L, Beyotime Biotechnology, Shanghai, China), the protein was denatured in a metal bath at 100°C for 10 min. The SDS-PAGE 10% or 15% separation gels were used to separate proteins, and then transferred proteins to hydrophobic polyvinylidene (PVDF) membranes and blocked with 5% non-fat dry milk. The primary antibodies were dropped to the PVDF membrane and incubated at 4°C for 12 h. The primary antibodies included Rabbit CitH3 (1:1500), Rabbit caspase-11 (1:1500, DF7609, Affinity), Rabbit GSDMD (1:1500, AF4012, Affinity), Rabbit CXCR-2 (1:1000, bs-1629R, Bioss), Rabbit MPO (1:1500, ab208670, Abcam) and Rabbit GAPDH (1:5000, YM3029, Immunoway, United States). A goat anti-rabbit lgG secondary antibody (ZB-2301, ZSbio, Beijing, China) was added dropwise to the PVDF membrane after being washed with TBST. After 1 min in dark, the EasySee Western Blot Kit (DW101-02, TransGen Biotech, Beijing, China) reacted with the PVDF membrane, and the bands were observed *via* the gel imaging system. The proteins expression levels of CitH3, CXCR-2, caspase-11, GSDMD and MPO were analyzed using ImageJ software and standardized to GAPDH (Shang et al., 2022).

### Immunohistochemistry assay

The paraffin sections of lung tissues were deparaffinized. After inhibiting endogenous peroxidase activity and repairing antigen, the non-specific binding sites were blocked with 10% BSA for 1 h. The sections were incubated with rabbit anti-NE and rabbit anti-CitH3 (1:200) for 12 h at 4°C and washed with PBST, followed by staining with secondary antibody for 40 min at 37°C. The color development was done with DAB kit (AR1022, Boster Bio, Wuhan, China), followed by hematoxylin staining and differentiation with 1% hydrochloric acid alcohol. Finally, the sections were sealed with neutral gum. The expressions of NE and CitH3 were detected with an optical microscope (Vectra 3) and quantified by ImageJ software (Shang et al., 2022).

### The measurement of neutrophils in sepsis mice by flow cytometry

#### Bronchoalveolar lavage fluid

In brief, the trachea of mice was intubated with syringe and flushed with PBS while the chest wall was tenderly massaged, and the BALF was obtained. Cells were resuspended in PBS after being centrifuged at 350 g for 5 min at 4°C. PE anti-mouse Ly6G (1:50, 127607, Biolegend) was used to label neutrophils. Then the samples were immediately analyzed with flow cytometry (Hook et al., 2019).

#### BM

As described (Mylonas et al., 2017), mouse femur was dissected and bone marrow cells were flushed out with syringe using PBS, after erythrocytes were lysed, the cells were centrifuged at 4°C, 350 g, 5 min to obtain leukocyte precipitation. Then the cells were resuspended in PBS, the percentage of neutrophil was detected by flow cytometry. FITC anti-mouse CD11b antibody (1:50, 101206, Biolegend) and PE anti-mouse Ly6G antibody (1:50, 108408, Biolegend) were used to stain neutrophils and then immediately analyzed with a flow cytometer.

#### Blood

As described (Souto et al., 2011), briefly, 24 h after CLP, blood was obtained by cardiac puncture and erythrocyte was lysed. The cells were centrifuged to obtain precipitated leukocytes, neutrophils were labeled with FITC anti-mouse CD11b (1:50, 101206, Biolegend) and PE anti-mouse Ly6G (1:50, 108408, Biolegend) antibodies. Then the cells were immediately analyzed with a flow cytometer.

### The isolation of neutrophils

We collected BM cells and filtered the suspensions with nylon mesh of 70 microns. On the gradient of Percoll (78%, 65%, 50%) solutions (Sigma, St. Louis, MO, United States), the cells were layered carefully and centrifuged at 800 g for 30 min. After centrifugation, neutrophils were in the “granulocytes” layer. The collected neutrophils were cultured in RPMI 1640 supplemented with 10% FBS and 1% penicillin and streptomycin (Hawez et al., 2019). The purity of neutrophils was >70%, which was determined with flow cytometry.

### Visualization of NETs and GSDMD-related genes using immunofluorescence assay

As described (Okeke et al., 2020), neutrophils were seeded in 96-well plate (Costar, Corning) with 2 × 10^5^/well and incubated in normal conditions for 3 h. After adherence, the cells were treated with 500 nM PMA (phorbol 12-myristate 13-acetate) and XBJ diluted with 1:50. After incubating for 6 h, the cells were fixed with 4% paraformaldehyde (PFA) and permeabilized with 0.5% Triton X-100. Then, the cells were stained with anti-NE (1:200), anti-CitH3 (1:200) and anti-MPO (1:200) primary antibodies for overnight at 4°C, and AlexaFluor 488-conjugated or 555-conjugated goat anti-rabbit IgG antibody (1:500; Abcam) was stained for 2 h in the dark. Hoechst 33342 (catalog no. H1399, United States) at 1 mg/ml was used to stain nucleus. Images were acquired using high-content imaging system (Operetta, Perkin-Elmer, United States).

Purified neutrophils were treated with 1.5 ug/ml LPS (L2880, Sigma) and 2.5 mM ATP (A7699, Sigma) to induce inflammatory death, then treated with XBJ. After cells were fixed and permeabilized, anti-caspase-11 (1:200) and anti-GSDMD (1:200) primary antibodies were incubated for overnight at 4°C, and then stained cells with AlexaFluor 488-conjugated goat anti-rabbit IgG antibody or PI (Propidium Iodide, 1:100, Solarbio, Beijing, China) for 2 h. Hoechst 33342 was used to stain nucleus. Images and analysis were acquired using high-content imaging system (Wang J. et al., 2022).

*In vivo*, 24 h after CLP, the neutrophils in lung tissues were isolated using the same methods as above experiments. Neutrophils were seeded in 96-well plate for 3 h to adhere. Then, the cells were stained with anti-caspase-11 and anti-GSDMD primary antibodies for overnight at 4°C, then AlexaFluor 647-conjugated goat anti-rabbit IgG antibody were incubated for 2 h. Hoechst 33342 was used to stain nuclei. Images were acquired using high-content imaging system.

### Lactate dehydrogenase detection in neutrophils

Purified neutrophils were seeded in 6-well plate (Costar, Corning) with 5 × 10^5^/well and incubated for 3 h. After adherence, the cells were induced with LPS and ATP, and then XBJ diluted for 1:50 was used to treat cells. The activity of lactate dehydrogenase (LDH) was detected using a kit (C0016, Beyotime Biotechnology) for detecting lytic cell death (Rathkey et al., 2018).

### Detection of MPO expression by flow cytometry *in vivo* and *vitro*

*In vitro*: the purified neutrophils were stimulated with PMA to induce NETs production and treated with XBJ for 6 h. After collecting cells, FITC anti-mouse MPO antibody (1:100, ab90812, Abcam) was used to dye cells for 40 min in the dark. The labeled cells were analyzed with flow cytometry (Thom et al., 2006).

*In vivo*: lung tissues were obtained at 24 h after CLP and were cut into small pieces and placed in digestion buffer containing 0.025 mg/ml collagenase I and 40 μg/ml DNase, and filtered the digested homogenization with 70 μm filter to get leukocytes. FITC anti-mouse MPO antibody was incubated for 40 min, immediately analyzed with flow cytometry.

### The application of GSDMD inhibitor (disulfiram) in mice and neutrophils

In isolated neutrophils, the cells were induced to form NETs by PMA and then treated with 10 μM disulfiram (CAS No.: 97-77-8, MedChemExpress) after cell adherence (Hu et al., 2020). Mice were treated with the disulfiram dissolved in sesame oil (12.5 mg/ml) *via* intraperitoneal injection for 50 mg/kg at 0 h, 12 h and 24 h after CLP.

### Statistical analysis

Log-rank tests were used to determine the statistical significance of Kaplan-Meier survival curves. ANOVA or unpaired t tests was performed on the remaining results, using the InStat software version 3.06 for Windows (GraphPad, San Diego, CA, United States). The statistical significance was expressed through the following terminology: *p < 0.05, **p < 0.01, ***p < 0.001 and ****p < 0.0001.

## Results

### XBJ ameliorated sepsis-induced lung injury and pulmonary dysfunction

**FIGURE 1:**
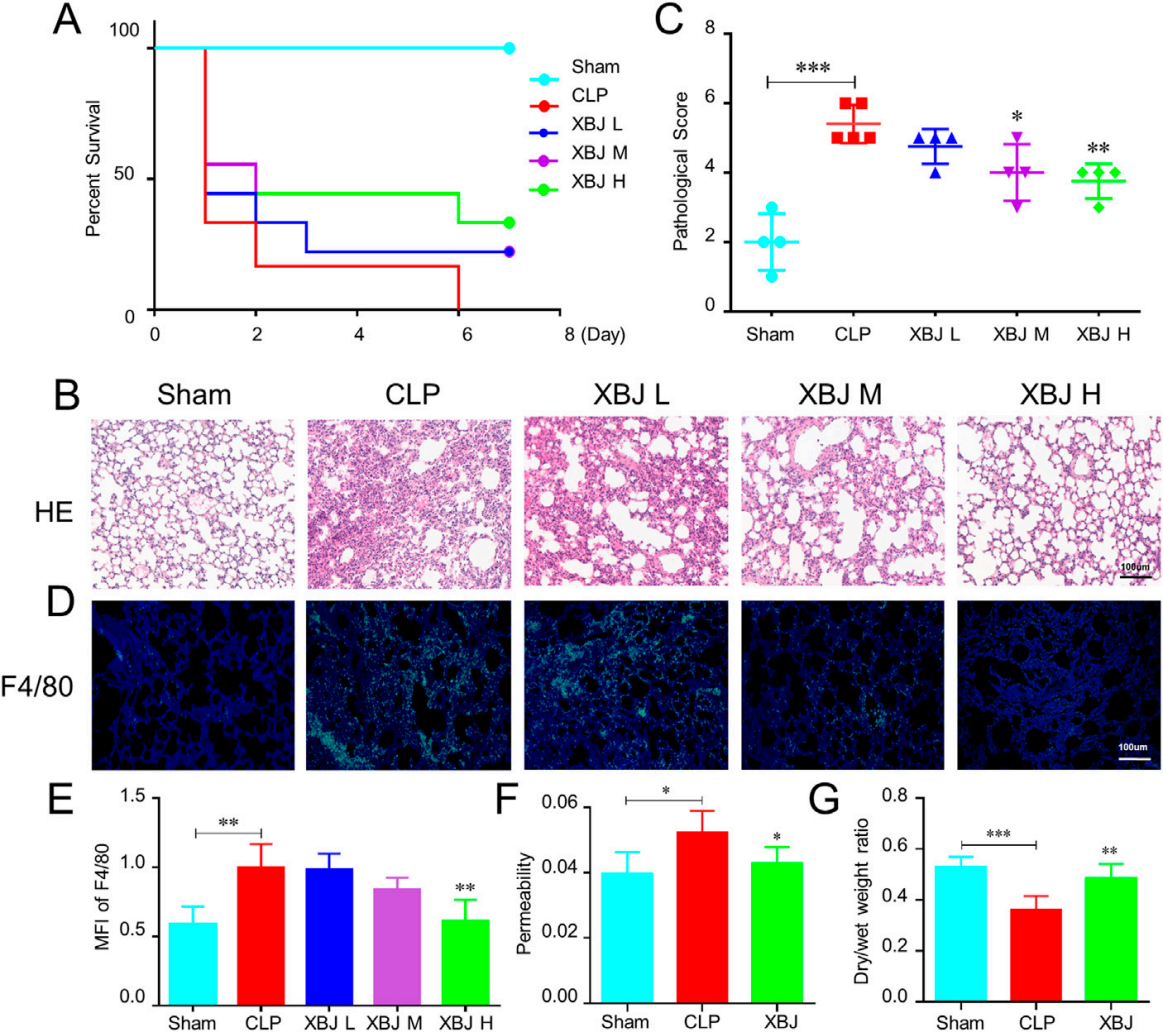
XBJ ameliorated sepsis-induced lung injury **(A)**. The Kaplan-Meier survival curve of different groups of mice. WT mice were subjected to CLP, then were injected (tail vein injection) with XBJ every 12 h in different concentrations (*n* ≥ 5 mice/group). Survival was monitored within 7 days. Lung tissues were collected from different group mice at 24 h after CLP **(B)**. The representative images of hematoxylin and eosin-stained lung tissues, which were observed under light microscopy at ×200 magnifications **(C)**. The pathological scores of lungs in different groups were quantified as described in Materials and Methods (*n* ≥ 3) **(D)**. Immunofluorescent assay to detect the macrophage (F4/80 positive) in the lung tissue, DAPI (blue) and F4/80 (green) were shown **(E)**. The quantitative result of F4/80 (*n* = 3). **(F,G)** showed the ratio of Evans blue dye extravasation and wet/dry weight ratio of lungs, respectively (*n* ≥ 5). The values were indicated as mean ± SD. *p < 0.05, **p < 0.01, ***p < 0.001, compared with the CLP group.

We adopted a murine model of septic shock by cecal ligation and puncture (CLP) to study the influence of XBJ on pulmonary tissue. The high dose of XBJ (18 ml/kg/day) improved the survival of CLP mice (Figure 1A). H&E staining was performed to assess the alteration of lung tissues at 24 h after CLP and we graded pathological scores following the established scoring criteria (Wang Y. et al., 2022). The high dose of XBJ treatment dramatically reduced the pathological deterioration caused by CLP, including edema, alveolar collapse, and inflammatory cell infiltration (Figures 1B,C). Macrophage infiltration contributes to the exacerbation of lung tissue, F4/80 fluorescent staining revealed that the high-dose XBJ alleviated the macrophage infiltration in lung tissues of septic mice (Figures 1D,E). Therefore, we conducted the follow-up experiments using high-dose XBJ treatment. Besides, the high dose of XBJ also improved the lung vascular permeability to albumin (Figure 1F) and reduced pulmonary edema (Figure 1G).

### XBJ reduced the neutrophil recruitment and chemokines in the lungs of septic mice

**FIGURE 2:**
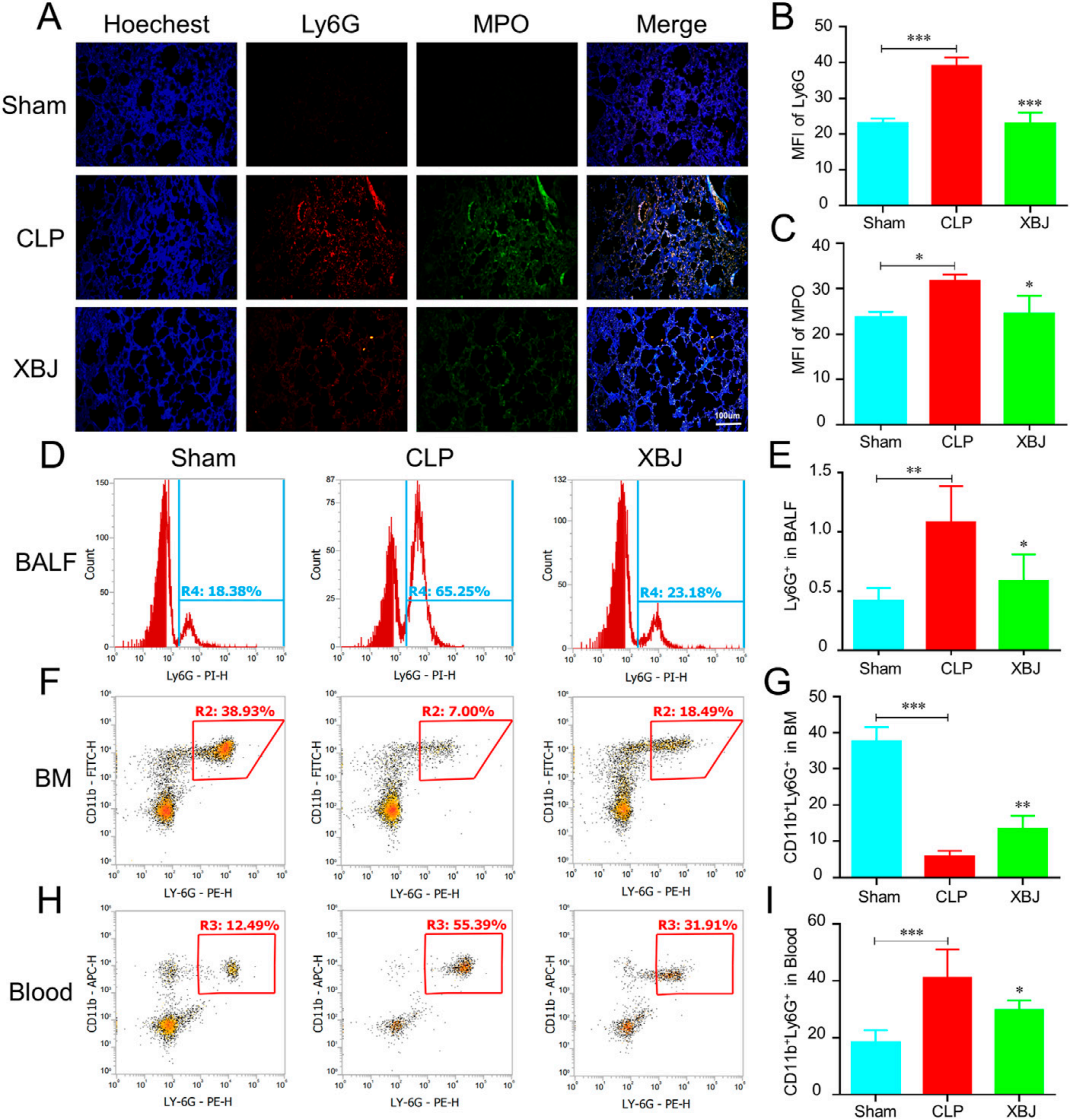
XBJ reduced the neutrophil recruitment to lung tissues in sepsis **(A)**. The confocal microscope images of Ly6G (red), MPO (myeloperoxidase, green) and nuclei (blue) staining in lung sections **(B,C)**. The quantification of Ly6G **(B)** and MPO **(C)** expression (*n* = 4) **(D,E)**. The Ly6G positive cells in bronchoalveolar lavage fluid (BALF) in different groups were analyzed with flow cytometry **(F,G)**. The neutrophil counts in bone marrow of septic mice were determined by flow cytometry using anti-CD11b and anti-Ly6G antibodies **(H,I)**. The percentages of CD11b and Ly6G double positive neutrophils in the peripheral blood of different groups of mice, which were determined with flow cytometry analysis. N = 5/per group. The values were indicated as mean ± SD. *p < 0.05, **p < 0.01, compared with the CLP group.

Neutrophil recruitment contributes to lung injury during sepsis. The immunofluorescent staining results showed there existed higher levels of Ly6G and MPO in the lungs of septic mice than sham mice, while XBJ treatment significantly reduced their expressions compared to the CLP group (Figures 2A–C). Consistently, the flow cytometry assay also uncovered that XBJ decreased the neutrophil counts in BALF induced by CLP (Figures 2D,E). To further confirm the influence of XBJ on neutrophil recruitment, we analyzed the neutrophil counts in the BM and peripheral blood. Intriguingly, there existed a higher percentage of CD11b^+^Ly6G^+^ neutrophils in the BM after XBJ treatment than CLP mice (Figures 2F,G). In line with the above results, XBJ significantly reduced the neutrophils in the peripheral blood (Figures 2H,I), demonstrating that XBJ could reduce neutrophil recruitment and infiltration to lung tissues in septic mice.

**FIGURE 3:**
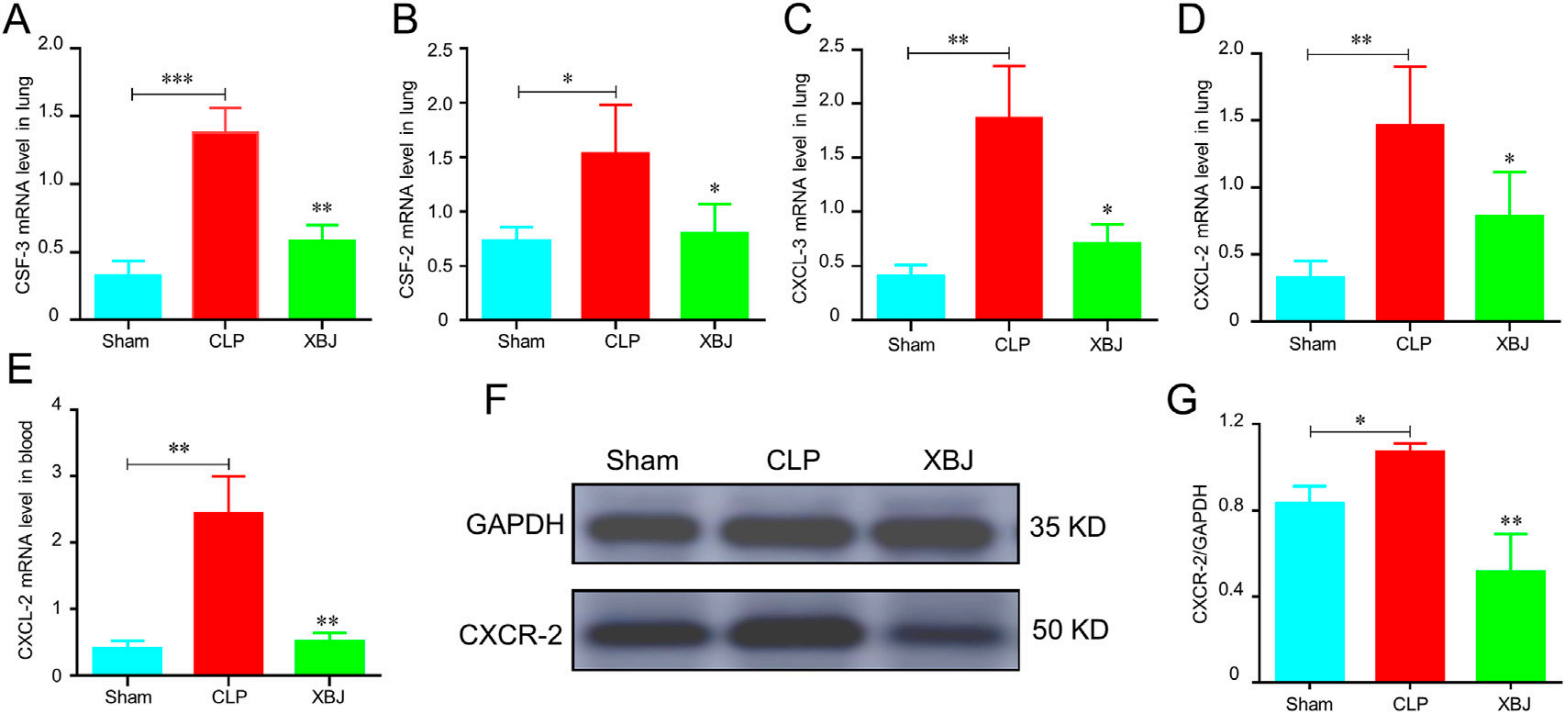
XBJ reduced the chemokines secretion in sepsis-induced lung injury. **(A–D)**. The relative mRNA expression levels of chemokines in lung tissues of different groups of mice, including CSF-3 **(A)**, CSF-2 **(B)**, CXCL-3 **(C)**, and CXCL-2 **(D)**, were determined by real-time PCR **(E)**. CXCL-2 mRNA expression in the peripheral blood of different groups of mice was determined by real-time PCR. N ≥ 4/per group. F&G. The CXCR-2 protein expression was determined with Western blot, the representative images were shown in **(F)** and were quantified in **(G)** (*n* = 3). The values were indicated as mean ± SD. *p < 0.05, **p < 0.01, compared with the CLP group.

Chemokines have crucial roles in neutrophil recruitment (Kolaczkowska and Kubes, 2013). As expected, the mRNA expressions of chemokines promoting neutrophil recruitment were significantly increased in the CLP group as compared with the sham mice, while XBJ normalized their expressions, including CSF-3, CSF-2, CXCL-3, and CXCL-2 (Figures 3A–D). Consistently, in our previous study, we demonstrated that XBJ significantly down-regulated the expression of CXCL-2 in sepsis-induced cardiac dysfunction (Wang et al., 2021). Furthermore, XBJ reduced CXCL-2 mRNA expression of leukocytes isolated from blood (Figure 3E). And XBJ also down-regulated the CXCR-2 protein expression in the lungs of septic mice (Figures 3F,G).

### XBJ reduced NETs formation in the lungs of septic mice

**FIGURE 4:**
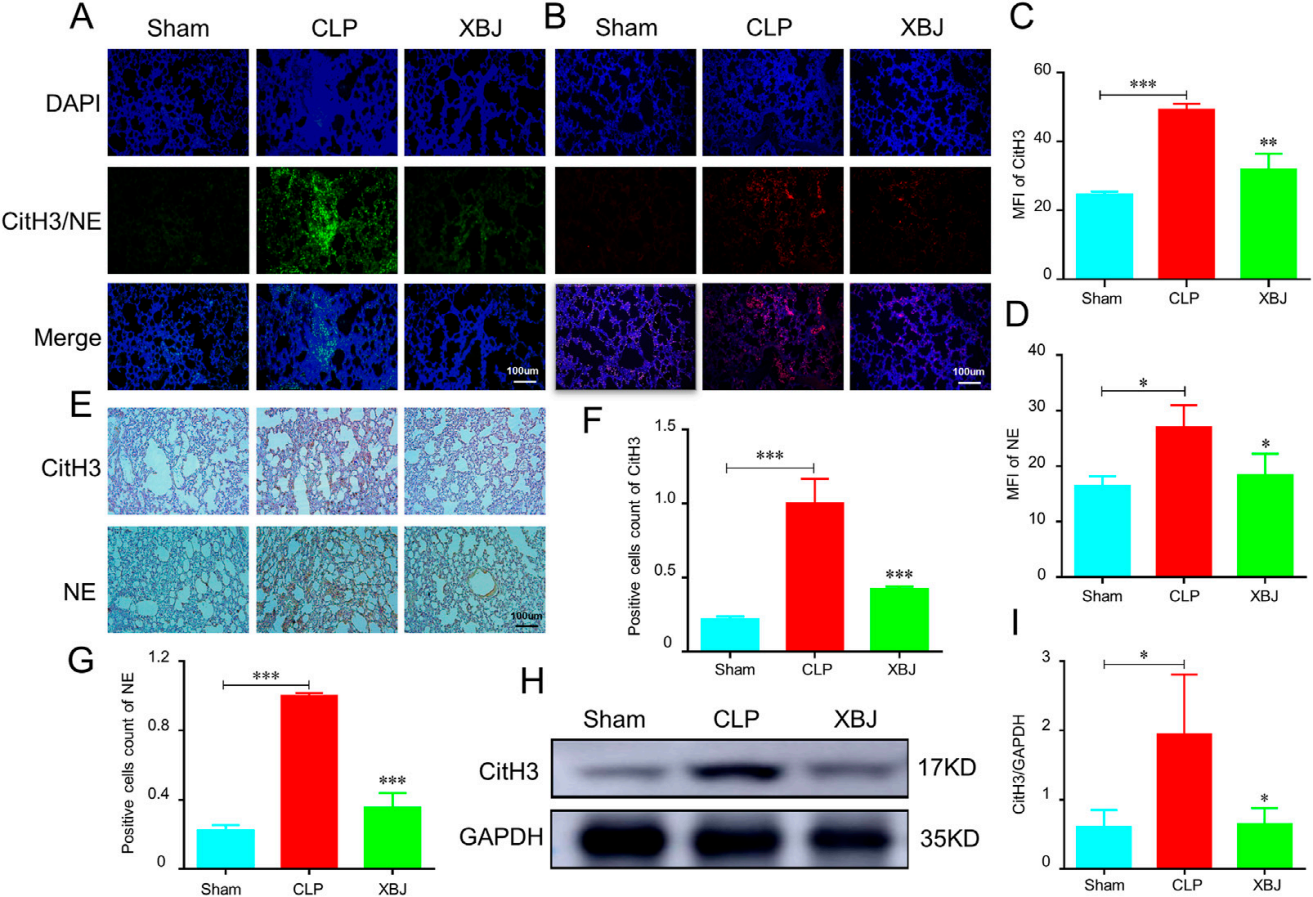
XBJ alleviated the NETs formation in the lungs of septic mice **(A–D)**. Immunofluorescent staining was performed on lung sections from CLP mice (*n* = 3) and analyzed by confocal microscopy. DAPI (blue), NE (Neutrophil elastase, red) and CitH3 (Citrulline histone 3, green) **(A,C)**. The expression of CitH3 in lung tissues of indicated groups of mice. The expression of NE in lung tissues of different groups of mice was shown in **(B,D–G)**. The expression of NETs formation markers, CitH3 and NE, were determined by immunohistochemistry in the lung sections of different groups of mice **(F,G)**. The quantifications of CitH3 **(F)** and NE **(G)** expression levels in immunohistochemistry assays (*n* = 3) **(H,I)**. Western blot was performed to determine the expression of CitH3 in the lung tissue of indicated groups of mice. I showed the quantification of CitH3 expression. N = 4/per group. The values were indicated as mean ± SD. *p < 0.05, **p < 0.01, ***p < 0.001, compared with the CLP group.

Excessive NETs formation is critical player in the development of thrombosis and organ failure during sepsis. Various components of NETs have been identified as initiators or propagators of organ dysfunction (Nicolai et al., 2020). Therefore, blocking NETs release would contribute to preventing lung injury. Consistent with other studies, we discovered the lung tissues of septic mice produce more CitH3 and NE, two markers of NETs (Burgener and Schroder, 2020) than the sham group, while XBJ effectively reversed their production in the immunofluorescent staining (Figures 4A–D). The immunohistochemical results also revealed that XBJ inhibited the expression levels of CitH3 and NE in lung tissues of septic mice (Figures 4E–G). These results were further confirmed with western blot analysis that XBJ significantly reduced the expression of CitH3 compared with the CLP group (Figures 4H,I).

**FIGURE 5:**
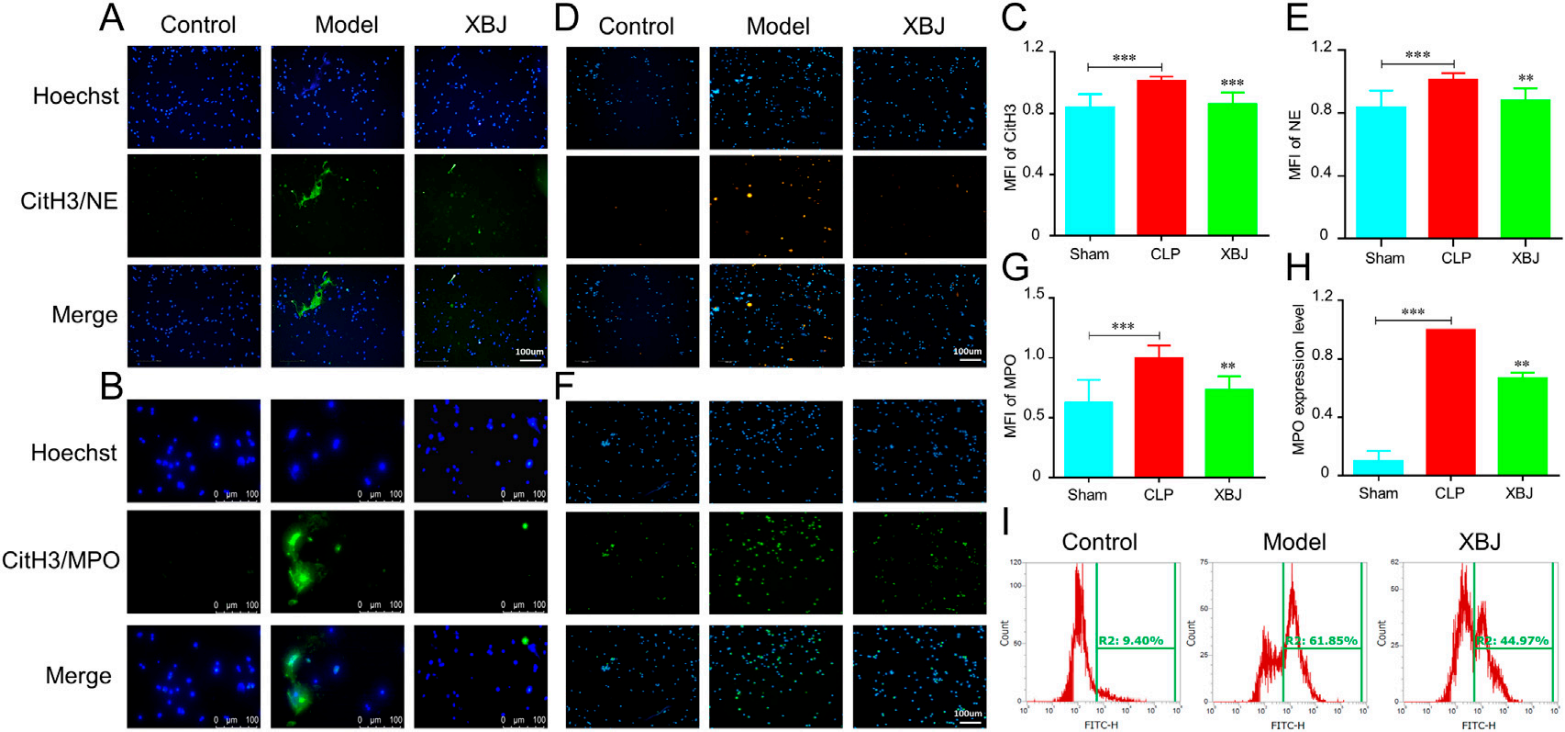
XBJ prevented the NETs production *in vitro*. The purified neutrophils from bone marrow were stimulated with PMA (phorbol 12-myristate 13-acetate) and incubated with/without XBJ **(A,B)**. NETs formation was detected by immunofluorescent staining for CitH3 (green) and hoechst (blue), the magnifications are ×200 **(A)** and ×400 **(B)** respectively **(C,D)**. NE **(C)** and MPO **(D)** expressions were determined by immunofluorescent assay to reveal the NETs formation, NE and MPO were shown with yellow and green, respectively **(E–G)**. The quantification of fluorescent intensity of CitH3 **(E)**, NE **(F)**, and MPO **(G–I)**. The expression of MPO in neutrophils was detected by flow cytometry, the representative images **(H)** and quantitative results **(I)** were shown. All experiments were repeated at least three times. The values were indicated as mean ± SD. *p < 0.05, **p < 0.01, ***p < 0.001, compared with the Model group.

To validate the above results, we evaluated the influence of XBJ on NETs formation *in vitro*. Importantly, XBJ treatment significantly inhibited CitH3 expression as compared with the model group (Figures 5A–C). The release of PMA-induced NE and MPO in neutrophils was also significantly decreased by XBJ in the immunofluorescent assay (Figures 5D–G). Consistently, XBJ treatment also effectively decreased the MPO expression in neutrophils detected by flow cytometry (Figures 5H,I).

### XBJ treatment normalized the sepsis-induced GSDMD over-expression

**FIGURE 6:**
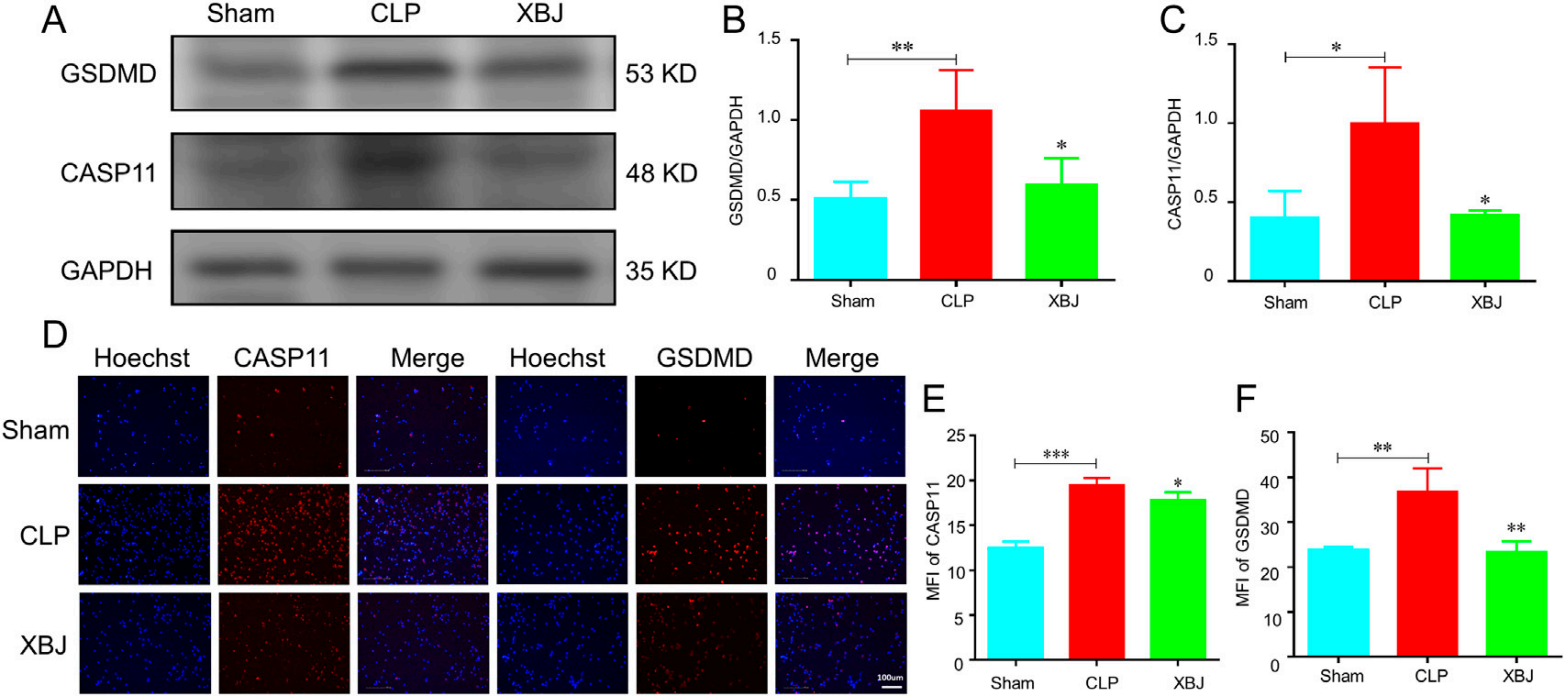
XBJ reduced the expression of caspase-11 and GSDMD in septic mice. The lung tissues were collected at 24 h after CLP. **(A–C)**. The expressions of GSDMD and caspase-11 were determined with Western blot **(A)** and quantified results **(B,C)**, respectively (*n* ≥ 3) **(D–F)**. The neutrophils in lung tissues were isolated and purified at 24 h after CLP. The expressions of caspase-11 and GSDMD were detected by immunofluorescent assay as described in the methods **(D)**. the representative images of immunofluorescent assays **(E,F)**. The quantification of caspase-11 **(E)** and GSDMD **(F)** expressions in neutrophils isolated from lung tissues. N > 3/group. The values were indicated as mean ± SD. *p < 0.05, **p < 0.01, ***p < 0.001, compared with the CLP group.

GSDMD, a pore-forming protein, plays a crucial role in the release of NETs in sepsis (Sollberger et al., 2018). The major components of XBJ, paeoniflorin and hydroxysafflor yellow A are identified can down-regulate GSDMD expression in depression mice and cerebral ischemia reperfusion models, respectively (Tan et al., 2020; Tian et al., 2021). Therefore, we wondered whether XBJ impacts the expression of GSDMD in sepsis-induced lung injury. As expected, significant inhibitory effects for GSDMD and caspase-11 expression was observed in the lung tissues after treatment with XBJ (Figures 6A–C). The immunofluorescent staining revealed that XBJ reduced sepsis-induced GSDMD and caspase-11 excessive expressions in neutrophils isolated from lung tissues (Figures 6D–F).

**FIGURE 7:**
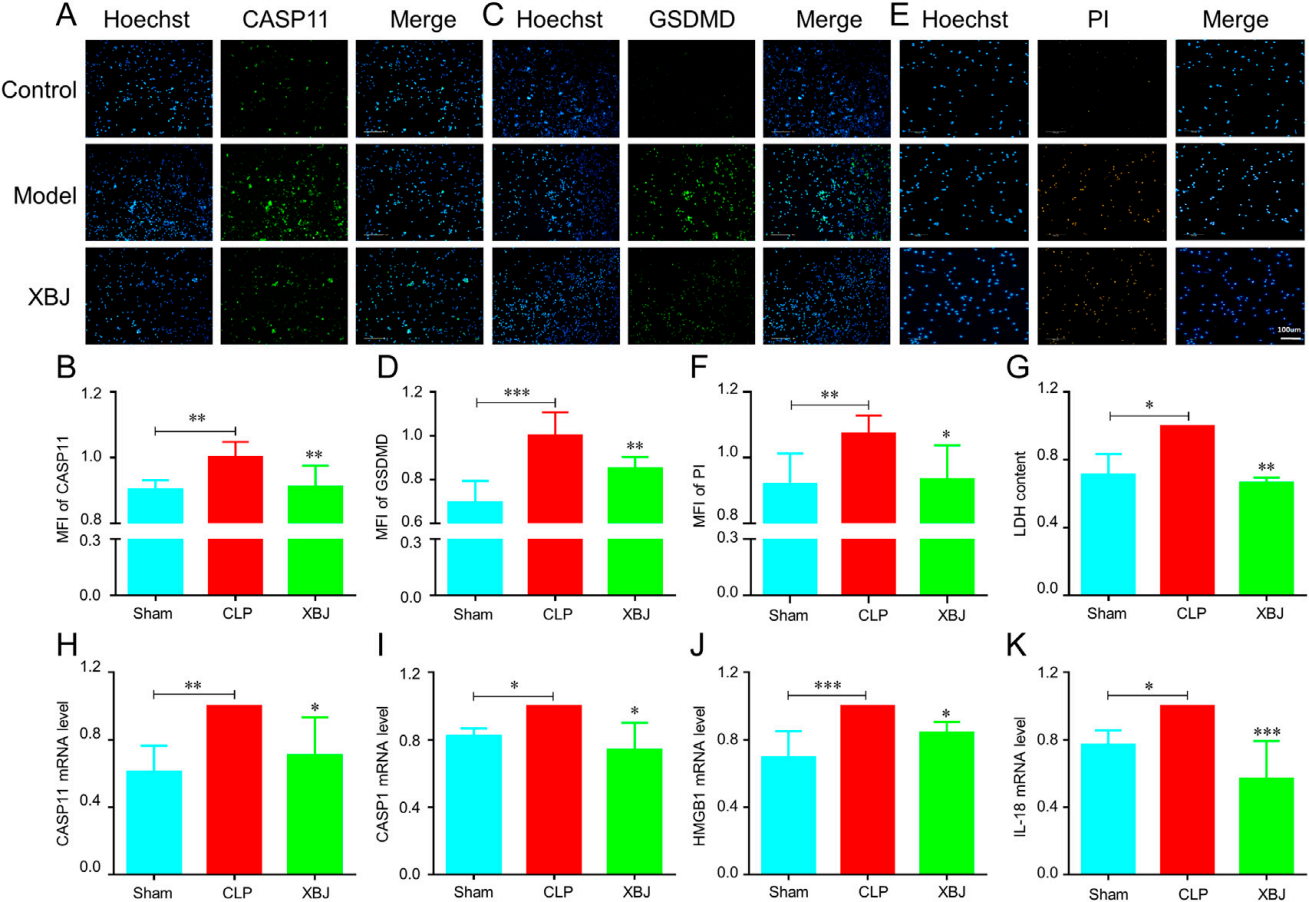
*In vitro*, XBJ reduced the expressions of GSDMD-related genes. The purified neutrophils from bone marrow were stimulated with 1.5 ug/ml LPS and 2.5 mM ATP and then incubated with or without XBJ **(A–F)**. The representative images and quantified results of caspase-11 **(A,B)** immunofluorescent staining, GSDMD **(C,D)** immunofluorescent staining, and PI staining **(E,F)** in the control, model, and XBJ treated groups **(G)**. The relative LDH expression in different groups of neutrophils **(H–K)**. The relative mRNA expressions of NETosis-related markers, caspase-11 **(H)**, caspase-1 **(I)**, HMGB1 **(J)**, and IL-18 **(K)** in neutrophils, were determined by real-time PCR. All experiments were repeated at least three times. The values were indicated as mean ± SD. *p < 0.05, **p < 0.01, ***p < 0.001, compared with the Model group.

Next, we investigated the influence of XBJ on the expressions of GSDMD and caspase-11 *in vitro*. Using immunofluorescent staining, we found that XBJ significantly inhibited the expressions of caspase-11 and GSDMD in neutrophils (Figures 7A–D). Then, in the PI staining assay, we also found that XBJ treated group showed fewer PI^+^ cells than the model group (Figures 7E,F). Besides, XBJ also decreased the LDH release in neutrophils (Figure 7G). We also determined the expression levels of upstream genes of GSDMD by RT-PCR assay and the results showed XBJ treatment effectively decreased the mRNA expressions of GSDMD upstream genes, including caspase-11, caspase-1, HMGB1, and IL-18 (Figures 7H–K). These results suggested that XBJ could inhibit sepsis-induced GSDMD and caspase-11 expressions.

### XBJ alleviated NETs formation by inhibiting GSDMD expression

**FIGURE 8:**
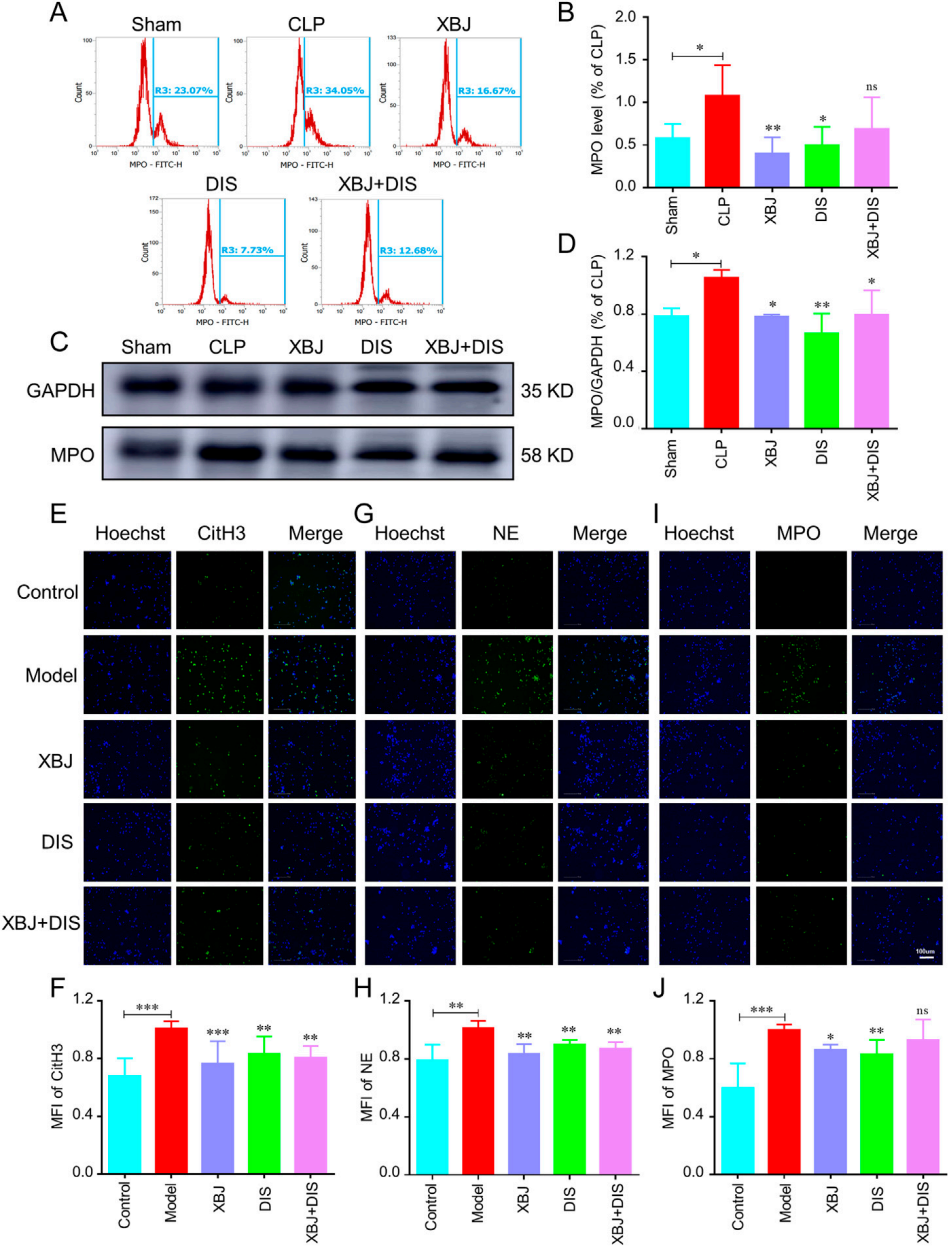
XBJ alleviated NETs formation by inhibiting GSDMD expression. Mice were treated with disulfiram (DIS) intraperitoneally after CLP (once every 12 h (50 mg/kg)), and the lung tissues were collected at 24 h, *n* ≥ 4 in each group. **(A, B)**. The percentage of MPO in lung tissues by flow cytometry. **(C, D)**. The MPO expression in lung tissues was determined with Western blot. The representative images of MPO **(C)** and statistical results **(D)** were shown. An immunofluorescent assay was used to determine the influence of XBJ and DIS on NETs formation. **(E‐J)**. The influence of XBJ on NETs formation was determined in vitro. Neutrophils harvested from mice were treated with 10 μM DIS, XBJ, and XBJ + DIS respectively. PMA was used to induce NETs formation. The expressions of CitH3 **(E, F)**, NE **(G, H)**, and MPO **(I, J)** were determined by immunofluorescent staining. The experiments were repeated at least three times. The values were indicated as mean ± SD. *p < 0.05, **p < 0.01, ***p < 0.001, compared with the Model group.

To further determine whether XBJ alleviated sepsis-induced NETs formation through the GSDMD signaling pathway, we used the GSDMD inhibitor, disulfiram (DIS) (Hu et al., 2020) to block its expression in CLP mice and neutrophils. Similar to XBJ, DIS reduced the over-expression of MPO in lung tissues of sepsis mice in flow cytometry and WB assays (Figures 8A–D), whereas, the XBJ + DIS treatment group did not show a remarkable difference compared with the DIS or XBJ treated group, suggesting that XBJ might reduce the NETs formation by inhibiting GSDMD. Consistent with above results, in isolated neutrophils, we also found DIS treatment group showed the low levels of NETs proteins including CitH3 (Figures 8E,F), NE (Figures 8G,H) and MPO (Figures 8I,J), and there was no significant difference between XBJ + DIS and DIS treatment groups. To sum up, we revealed XBJ reversed NETs formation by reducing GSDMD excessive expression induced by sepsis.

## Discussion

### Summary of the results

Sepsis is one of the most unaffordable medical conditions in hospitals that may lead to life-threatening MODS. The lung is the most frequent organ to fail. There is a clear link between NETs release and ARDS, and targeting NETs formation is an effective management in easing sepsis-induced lung injury (Abrams et al., 2019). However, there are currently no specific treatments for this syndrome, and novel therapeutic strategies need to be developed. Previous researches have revealed XBJ can alleviate the clinical symptoms of sepsis and COVID-19 patients (Huang et al., 2021), but there are no enough animal studies to reveal its mechanisms in depth. Therefore, in this work, we aimed to reveal the mechanisms of XBJ in a clinically relevant sepsis model. We found that XBJ protected sepsis-induced lung injury by blocking NETs formation. Additionally, we revealed that XBJ regulated NETs formation by restraining the GSDMD-mediated neutrophil death.

Firstly, we found that XBJ prevented lung injury caused by sepsis (Figure 1). Mechanistically, we investigated the effects of XBJ on neutrophil recruitment during sepsis. XBJ not only prevented neutrophil infiltration in the lungs (Figures 2A–E) but also decreased neutrophils in blood (Figures 2H,I). Consistently, XBJ retained neutrophil in the bone marrow (Figures 2F,G). Consistent with our results, other reports also revealed XBJ protect against lung injury induced by sepsis *via* down-regulating leukocyte adhesion (Zhang et al., 2018). Additionally, He et al. (2018) also found XBJ reduced neutrophil counts in BALF and ameliorated lung injury caused by dichlorvos poisoning. XBJ also can reduce the activities of MPO and NE in multiple organ tissues after burn injury (Tang et al., 2017). Chemokines contribute to the neutrophil recruitment, and we further confirmed that XBJ significantly decreased the expressions of inflammatory chemokines including CSF-3, CSF-2, CXCL-3, CXCL-2 and CXCR-2 (Figure 3).

When sepsis occurs, activated neutrophils release NETs, contributing to lung injury and pulmonary coagulation. Intravascular NETs-induced coagulation results in MODS in sepsis (Thom et al., 2006). Wang et al. (2020); Zha et al. (2021) uncovered that hydroxysafflor yellow A and senkyunolide I, the main components in XBJ, prevent lung injury *via* inhibiting the formation of NETs in lung injury induced by LPS and CLP, respectively. Hence, we wondered whether XBJ can inhibit NETs production in sepsis. Strikingly, the results of immunohistochemistry, immunofluorescence and WB assays revealed that XBJ is sufficient to obstruct the NETs formation (Figure 4). In isolated neutrophils, we further confirmed the above results *in vitro* (Figure 5). Based on these results, we concluded that XBJ inhibited sepsis-induced NETs formation.

In the process of sepsis, GSDMD is required for the formation of NETs, a special form of neutrophil death that releases chromatin structures into the extracellular space (Sollberger et al., 2018; Yang et al., 2019). Some studies demonstrated that hepatocyte-released HMGB1 played a vital role in mediating caspase-11-dependent pyroptosis and sepsis lethality. Previous research uncovered that XBJ can down-regulate HMGB1 expression induced by sepsis (Liu et al., 2016; He et al., 2018). And XBJ also reduced IL-1β expression level, the upstream factor of caspase-11 and GSDMD (Wang et al., 2021). Therefore, we evaluated the expression levels of caspase-11 and GSDMD in lung tissues of septic mice by WB assay. As expected, XBJ reduced their over-expressions in sepsis-induced lung injury (Figures 6A–C). Uniformly, XBJ also inhibited the caspase-11 and GSDMD expressions in neutrophils isolated from lung tissues of septic mice (Figures 6D–F). Simultaneously, *in vitro*, caspase-11, GSDMD protein expressions, and the mRNA levels of upstream genes of GSDMD were all reduced by XBJ treatment (Figure 7).

These results suggested that XBJ might alleviate NETs formation by inhibiting GSDMD expression. To verify the above hypothesis, DIS was used to block GSDMD expression in sepsis mice and neutrophils, we found that disulfiram can significantly reduce NETs production (Figure 8). It was worth noting that the XBJ + DIS treatment group also alleviated NETs formation, but there existed no significant difference when compared with DIS treatment group (Figures 8A–F). These results indicated that XBJ alleviated NETs production caused by sepsis through inhibiting GSDMD over-expression. In agreement with our results, a recent report also found that DIS down-regulate complement/coagulation pathways and reduce NETs formation, thereby protecting rodents from acute lung injury (Adrover et al., 2022).

## Conclusion and future direction

In this report, we explored the pharmacological mechanism of XBJ in sepsis-induced lung injury. We revealed that XBJ alleviated the lung injury and neutrophil recruitment caused by sepsis. We further demonstrated that XBJ inhibited GSDMD-related signaling pathway to reduce the pulmonary NETs production in septic mice. These results partially explained the clinical effectiveness of XBJ. However, there are other signaling pathways that mediate NETs formation in sepsis except GSDMD-mediated pathway, such as oxidative stress (McDowell et al., 2021) and type I interferon signaling (Yang et al., 2020), It is not clear whether XBJ impact these pathways in neutrophils. In addition, we also need to identify the main compounds in XBJ that impact the NETs formation. Recent studies revealed that NETs provide a scaffold to recruit red blood cells, platelets, as well as bind plasma proteins (Mao et al., 2021). The aggregation of NETs in the coronary arteries contributes to the growth and stability of the thrombus (McDonald et al., 2017). Therefore, the influence of XBJ for the interactions between NETs and coagulation in sepsis also need to be revealed.

XBJ has been used as an effective medicine for COVID-19 in China, but its detailed mechanism is still unclear. Some researches reveal that the levels of NETs markers are obviously increase in serum of COVID-19 patients (Masso-Silva et al., 2022) and thrombotic complications are frequent and contribute to a higher mortality (Connors and Levy, 2020). Besides, SARS-CoV-2 virus directly activates the GSDMD, which induce the NETs release and lung injury in COVID-19 (Silva et al., 2022). Based on our studies, it was possible that XBJ cure COVID-19 patients effectively by inhibiting NETs production, which was achieved by restraining GSDMD over-expression.

## Data Availability

The original contributions presented in the study are included in the article/supplementary materials, further inquiries can be directed to the corresponding authors.
